# Impact of Experimentally Induced Cognitive Dietary Restraint on Eating Behavior Traits, Appetite Sensations, and Markers of Stress during Energy Restriction in Overweight/Obese Women

**DOI:** 10.1155/2018/4259389

**Published:** 2018-06-25

**Authors:** Isabelle Morin, Catherine Bégin, Julie Maltais-Giguère, Alexandra Bédard, André Tchernof, Simone Lemieux

**Affiliations:** ^1^Institute of Nutrition and Functional Foods (INAF), Pavillon des Services, Laval University, 2440 Boulevard Hochelaga, Québec, QC, Canada G1V 0A6; ^2^School of Nutrition, Pavillon Paul-Comtois, Laval University, 2425 Rue de l'Agriculture, Québec, QC, Canada G1V 0A6; ^3^School of Psychology, Pavillon Félix-Antoine-Savard, Laval University, 2325 Rue des Bibliothèques, Québec, QC, Canada G1V 0A6; ^4^Endocrinology and Nephrology, CHU de Québec, Laval University Medical Center, 2705 Boulevard Laurier, Québec, QC, Canada G1V 4G2; ^5^Quebec Heart and Lung Institute, Laval University, 2725 Chemin Ste-Foy, Québec, QC, Canada G1V 4G5

## Abstract

Weight loss has been associated with changes in eating behaviors and appetite sensations that favor a regain in body weight. Since traditional weight loss approaches emphasize the importance of increasing cognitive dietary restraint (CDR) to achieve negative energy imbalance, it is difficult to untangle the respective contributions of energy restriction and increases in CDR on factors that can eventually lead to body weight regain. The present study aimed at comparing the effects of energy restriction alone or in combination with experimentally induced CDR on eating behavior traits, appetite sensations, and markers of stress in overweight and obese women. We hypothesized that the combination of energy restriction and induced CDR would lead to more prevalent food cravings, increased appetite sensations, and higher cortisol concentrations than when energy restriction is not coupled with induced CDR. A total of 60 premenopausal women (mean BMI: 32.0 kg/m^2^; mean age: 39.4 y) were provided with a low energy density diet corresponding to 85% of their energy needs during a 4-week fully controlled period. At the same time, women were randomized to either a condition inducing an increase in CDR (CDR+ group) or a condition in which CDR was not induced (CRD− group). Eating behavior traits (Three-Factor Eating Questionnaire and Food Craving Questionnaire), appetite sensations (after standardized breakfast), and markers of stress (Perceived Stress Scale; postawakening salivary cortisol) were measured before (*T* = 0 week) and after (*T* = 4 weeks) the 4-week energy restriction, as well as 3 months later. There was an increase in CDR in the CDR+ group while no such change was observed in the CDR− group (*p*=0.0037). No between-group differences were observed for disinhibition, hunger, cravings, appetite sensations, perceived stress, and cortisol concentrations. These results suggest that a slight increase in CDR has no negative impact on factors regulating energy balance in the context of energy restriction.

## 1. Introduction

The treatment of obesity has become a public health priority given the negative impact of this condition on physical (i.e., cardiovascular diseases, type 2 diabetes, cancer, and musculoskeletal diseases) [1] and mental (i.e., body image disturbance and poor self-esteem) [2] health. However, despite many efforts from patients and healthcare professionals, long-term success in weight loss remains relatively scarce [3]. In fact, the most optimistic studies report that no more than 20% of people who lost at least 10% of their initial body weight are able to maintain the weight loss for more than one year [3].

Several studies addressing the issue of poor long-term success in weight loss maintenance have suggested that metabolic adaptations that occur in response to energy restriction can favor a return toward initial body weight [4, 5]. In response to the state of energy imbalance, body fat stores are gradually depleted and many hormonal signals are secreted to promote appetite in order to increase energy intake and replenish body fat stores [6]. Changes in appetite-regulating hormones, such as ghrelin and leptin, have been found to enhance the drive to eat in response to an energy-restricted state [6, 7]. Concomitantly with the occurrence of hormonal changes, studies have documented that appetite sensations are increased in an energy-restricted state [6, 7]. At the same time, a decrease in the metabolic rate is also observed in response to weight loss, favoring weight regain [4].

In order to counteract these homeostatic factors, traditional weight loss interventions frequently include behavioral strategies that encourage people to engage in cognitive control over the food they are eating [8]. People on traditional weight loss diet are thus trained to use cognitive strategies to control food intake such as counting calories, limiting high energy density food, and reducing portion size [9, 10]. This conscious control of eating behavior that is often referred to as cognitive dietary restraint (CDR) is in line with the definition proposed by Stunkard and Messick when they developed the Three-Factor Eating Questionnaire (TFEQ) [11]. Other instruments are available to measure CDR but the construct measured varies according to the instrument used. For example, the Restraint Scale was developed by Herman and Mack to identify chronic dieters who are highly preoccupied with their weight [12], which is different from what is evaluated by the CDR factor of the TFEQ. It is important to emphasize that most weight-loss studies which have documented the association between changes in CDR and body weight changes have used the TFEQ to measure CDR [13–16].

Increasing CDR through intervention strategies has been frequently associated with more successful weight loss in various populations [17, 18], and some authors have proposed that increasing CDR level is an adaptive strategy necessary to prevent weight regain driven by homeostatic factors in a food environment characterized by overabundance [18]. However, although increasing CDR is a suggested predictor of successful weight loss in traditional approaches, it has also been observed that efforts to keep CDR high can be counterproductive as demonstrated by longitudinal studies showing that a high CDR at baseline is characterized by greater weight regain during the follow-up [19, 20]. This could be explained by the fact that increased CDR has been associated in the literature with obsessive thoughts about forbidden foods [21], increased appetite sensations [22], and risk of overeating episodes (i.e., disinhibition) [23]. A high CDR has also been associated with increased stress [24–27]. This increase in stress level can be explained by the fact that self-regulation efforts required to control body weight can be stressful on a daily basis [28, 29]. Since many studies have underlined a positive association between stress and overeating [30–33], it is possible that increased stress associated with CDR could be one of the central mechanisms through which induced CDR can lead to a weight regain.

According to current evidence, it is clear that the association between induced CDR and factors controlling energy balance is extremely complex. Since most studies performed to date have been conducted in a context favoring both energy restriction and induced CDR, it is difficult to untangle the respective contributions of energy restriction (i.e., homeostatic factors) and induced CDR (i.e., cognitive factors) on variables that can lead to weight regain. Therefore, the overall objective of the present study is to compare the effects of energy restriction alone or in combination with induced CDR on eating behavior traits, appetite sensations, and markers of stress in overweight and obese premenopausal women. We hypothesized that women in the energy-restriction-plus-induced CDR condition have more prevalent food cravings, increased appetite sensations, and higher cortisol concentrations than women in the energy-restriction-without-induced CDR condition.

## 2. Subjects and Study Design

### 2.1. Subjects

This study was conducted among a sample of premenopausal women aged between 26 and 50 years and recruited through different media advertisements in the Québec City area, Canada. Subjects were considered as eligible if they had a body mass index (BMI) > 25 kg/m^2^, a stable body weight (±2.5 kg) for the last 3 months prior to the study, and a premenopausal status. If needed, a follicle-stimulating hormone (FSH) measurement was performed (e.g., when women presented period irregularities) to confirm the premenopausal status (FSH < 20 IU/L) [34]. Women were excluded if they had endocrine disorders, cardiovascular diseases, or type 2 diabetes; were taking medication that could affect dependent variables under study (namely medication affecting appetite); were a smoker, pregnant, or lactating; had any food allergies or food aversions that could impede compliance to the diet; or had psychiatric [35] or eating disorders [36]. Using GLMpower procedure and an attrition rate of 10%, a sample size of 58 women (29 in each of the two experimental conditions) was considered as sufficient to detect a 30% difference in cortisol concentrations and a 30% difference in appetite sensations between the two groups at the end of the intervention (SD = 40%, alpha = 0.05 (1-sided), power = 0.85). This study was conducted according to the guidelines laid down in the Declaration of Helsinki and all procedures involving human subjects were approved by the Laval University Ethics Committee (reference no. 2013-170/27-09-2013). This clinical trial was registered at www.clinicaltrials.gov (NCT02230111).

### 2.2. Study Design

The 4-week intervention was undertaken as a parallel design and conducted in 4 phases (from January 2014 to December 2014). Women were randomized to either an energy-restriction-plus-induced CDR condition (CDR+ group) or an energy-restriction-without-induced CDR condition (CDR− group). The 4-week intervention was followed by a three-month free-living period after which participants came back for a follow-up appointment (*T* = 16 weeks). Women were tested before (*T* = 0 week) and after (*T* = 4 weeks) the 4-week intervention, and at the follow-up visit (*T* = 16 weeks).

In order to appropriately test our hypotheses, participants were not informed of the real purpose of the study. Therefore, the cover study was to test the impact of a diet rich in fruits and vegetables on cardiovascular health. Women were debriefed about the real purpose of the experiment only at the end of the study. All women reported to have no idea of the real purpose before the debriefing.

#### 2.2.1. Energy Restriction

During the 4-week intervention, all women from the two experimental conditions (CDR+ and CDR− groups) were provided with the same reduced-calorie, low energy density diet which accounted for 85% of their energy needs (details of the diet composition are provided in Table 1). Even if energy intake was variable from one woman to another, the diet composition as presented in Table 1 was constant for all energy intake levels. Previous studies have shown that a low energy density diet is more satiating than a higher energy density diet in the context of caloric restriction [37, 38]. The diet used for the present study had to be satiating to ensure that participants in the CDR−group would not detect the caloric restriction, a situation which could have induced an increase in their CDR. Based on previous studies, a 15% energy restriction was used since this level of energy restriction could be achieved without increased feelings of deprivation in the context of a low energy density diet [39, 40].

To ensure that the caloric restriction induced in both groups was the same, all foods and drinks were prepared by food technicians at the Clinical Investigation Unit (CIU) at the Institute of Nutrition and Functional Foods (INAF, Laval University) and were provided to participants according to a 7-day cyclic menu (Supplementary Material, Table S1). On weekdays, participants came to the CIU to consume their noon meal under supervision, at which time they also received their evening meal and the next day's packaged breakfast to take home. Weekend meals were prepared, packaged, and provided at the CIU on the Friday visits. Every participant was instructed to consume all foods provided and to record their daily food consumption using a checklist. The difference between what was provided to participants and the proportion of food they reported eating was then computed, which was used as an indicator of compliance to the diet. More precisely, women were asked to report for each item of a meal whether they consumed it or not. If they partially ate a food item they had to indicate the percentage of the portion consumed. To calculate compliance, each food item of a meal was scored. If the food was totally consumed, a score of 1 was attributed whereas a score of 0 was attributed if the food item was not consumed at all. A score between 0 and 1 would be attributed for partially consumed portions (e.g., score of 0.5 if half of the portion was consumed). An integrated score including all food consumed during the 4-week intervention was then computed and expressed in percentage. Even if the consumption of nonstudy food was prohibited, participants were also instructed to detail any consumption of nonprovided food on their record, if any.

Prior to the beginning of the intervention, energy needs were determined for each woman using resting metabolic rate (RMR) measurement. RMR was assessed in the fasting state by indirect calorimetry (mouthpiece and nose-clip protocol), using a MOXUS Modular VO2 System (AEI Technologies, Naperville, IL). Women lied supine in a comfortable position and were asked to breathe normally through a Hans-Rudolph mouthpiece with nose-clip. VO2 and VCO2 (and thus RMR) measures were collected as breath-by-breath samples and averaged at 30-second intervals for 15 minutes. A 7-day physical activity record was also completed by each participant to determine an activity factor [41]. The measured RMR was multiplied by the activity factor to obtain total energy expenditure from which a 15% subtraction was applied to determine targeted energy intake to induce a slight reduction in body weight. Weight was monitored throughout the intervention in order to ensure that we successfully created an energy deficit. A predicted weight loss was calculated for each participant and, if women did not lose enough weight or if they lost more weight than expected, energy was readjusted during the intervention.

Expected weight loss was computed using the value of energy restriction calculated for each woman. More precisely, we used calculated energy needs (Table 2) and applied a 15% subtraction to this value. For example, a woman with estimated energy needs of 10,000 kJ/d would have an energy restriction of 1,500 kJ/d. Considering that a restriction of approximately 32,200 kJ is needed to lose one kg of body weight [42, 43], we were able to calculate the expected body weight loss per week. Members of the research team (IM, JMG, and SL) met every week to discuss each case. Body weight trajectories were examined and it was then decided to either keep the same energy intake or to make some changes (increases or decreases) in the energy intake. Increases or decreases of 523 kJ (125 kcal) or 1,046 kJ (250 kcal) per day offered to women through our controlled feeding protocol could be applied, depending on the magnitude of the difference between expected and actual weight change. Besides body weight values, more subjective elements such as feedback from the participants and the experience of the research team with controlled feeding studies (in which body weight control is essential to appropriately isolate the impact of the diet) [44–47] were also used in making the decision about changes to be made or not in energy intake of our participants.

#### 2.2.2. CDR Manipulation

Specific information about the diet was provided by a registered dietitian (RD) to each participant according to their experimental condition during the first testing visit (*T* = 0 week). In addition, throughout the 4-week intervention, messages were provided individually by the RD in order to reinforce the experimental condition setting.

In the CDR+ group, participants were told that the diet was hypoenergetic and the focus was on the importance of achieving weight loss during the intervention in order to enhance the benefits of the diet. Frequent weighing has been associated with an increase in CDR [48], and some studies also suggest that using traditional weight-centered dietary messages, even in the context of small energy restriction, is effective to induce an increase in CDR [49, 50]. Thus, participants in the CDR+ group were weighed every weekday when they came to the laboratory during lunch time and the importance of losing weight was repeated to women throughout the intervention. In the CDR+ group, women were also informed when the research team had to decrease the amount of food offered if they did not lose enough weight but were not notified when their energy intake was increased if they lost more weight than expected.

In the CDR− group, strategies previously developed by our research group to avoid restrictive messages were used [49]. Women were told that the diet was isoenergetic and the focus was exclusively on the importance of fruit and vegetable intakes for cardiovascular health. Participants were only weighed twice a week and were told that this was done in order to appropriately adjust their diet if needed. No emphasis was placed on weight loss but, if a participant noticed it, she was told that weight could normally fluctuate from day to day. Contrary to CDR+ group, women from the CDR− group were informed when the research team had to increase the amount of food offered if they lost more weight than expected but were not notified when their energy intake was decreased if they did not lose enough weight.

After the 4-week intervention, all participants met a RD to receive further information and advice to incorporate more fruits and vegetables in their usual diet. In the CDR+ group, the RD also advised participants to pursue their weight loss on their own and keep on with strategies relying on CDR. For example, they were told about how to decrease added sugar and fat in their diet and were provided a list of food whose consumption should be limited. Those strategies have been previously associated with an increase in CDR in participants in weight loss interventions [48, 49]. In the CDR− group, the RD used strategies developed in the context of a previous study to appropriately convey nonrestrictive messages [49]. Moreover, participants were taught about the importance to listen to their appetite sensations. The focus was also put on the concept of low energy density diet and its positive impact on cardiovascular diseases.

### 2.3. Measurements

#### 2.3.1. Anthropometric Profile

Women were weighed (within 0.1 kg; BWB-800 digital scale, Tanita) without shoes and while wearing light clothing on a calibrated scale at each testing visit (*T* = 0 week, *T* = 4 weeks, and *T* = 16 weeks). Standing height was measured at *T* = 0 week to the nearest millimeter. Body mass index (BMI) was then calculated according to standardized procedures [51]. Waist and hip circumference measures were also taken to the nearest millimeter according to standardized procedures [51] at each testing visit.

#### 2.3.2. Eating Behaviors Questionnaires

The Three-Factor Eating Questionnaire (TFEQ) [11] is a 51-item validated questionnaire that assesses three factors that refer to cognitions and behaviors associated with eating: CDR (cognitive control of eating behavior), disinhibition (the loss of control over eating in response to emotional or social cues), and hunger (food intake in response to feelings and perceptions of hunger). Rigid restraint and flexible restraint are two specific subscales that can also be derived from the general CDR score [52]. The trait version of the Food Craving Questionnaire (FCQ-T) [53], which is a 39-item questionnaire assessing how cravings are manifested in any given individual, was also completed by women. These questionnaires were completed at each testing visit (*T* = 0 week, *T* = 4 weeks, and *T* = 16 weeks).

#### 2.3.3. Appetite Sensations

Measurements of appetite sensations were performed following a standardized breakfast at *T* = 0 week and *T* = 4 weeks, as previously described [54]. The fact that appetite sensations reflect both objective (i.e., physiological) and subjective (i.e., learned) dimensions of appetite control makes their use relevant in the context of our study [55]. In fact, energy restriction (physiological) and CDR (learned) were manipulated in our study and could both influence appetite sensations. Studies have shown that appetite sensations measured during a standardized breakfast are predictive of subsequent energy intake and weight loss [56, 57]. In addition, measuring appetite sensations in response to a standardized breakfast is a methodology that has previously been used to study metabolic adaptations to weight loss [6].

Breakfast was served after a 12 h fast and contained white bread (two slices), peanut butter (18 g), butter (20 g), cheddar cheese (21 g), and orange juice (250 ml). The caloric content of the standardized breakfast was 2,749 kJ, and women were instructed to consume all the food provided. Women were invited to rate their desire to eat, hunger, fullness, and prospective food consumption (PFC) according to 4 visual analog scales (VAS) ranging from 0 to 150 mm. Appetite sensations were recorded before, immediately after, and every 10 minutes for 1 h after breakfast consumption. Questions were asked as follows: How strong is your desire to eat? (very weak to very strong); How hungry do you feel? (not hungry at all to as hungry as I ever felt); How full do you feel? (not full at all to very full); How much food do you think you could eat? (nothing at all to a large amount). In order to integrate all dimensions of appetite sensations into a unique indicator, we used an integrated appetite score (AS) as originally described by Anderson et al. [58]:

(1) $$\begin{array}{c} \text{AS}=\frac{{\text{VAS}}_{\text{hunger}}+{\text{VAS}}_{\text{desire to eat}}+{\text{VAS}}_{\text{PFC}}+\left(150-{\text{VAS}}_{\text{fullness}}\right)}{4}. \end{array}$$

One-hour postmeal area under the curve (AUC) in response to the standardized breakfast was also calculated for all appetite sensations, according to the trapezoid method, as described by Doucet et al. [59].

As proposed by Das et al. [50], overall level of hunger, desire to eat nonstudy foods, and satisfaction with the amount of food consumed were rated at the end of every intervention day on a 150 mm five point anchored VAS. The descriptors for each point were, for example, “not at all hungry,” “slightly hungry,” “moderately hungry,” “very hungry,” and “extremely hungry.” The participants marked a vertical line along the scale to indicate their overall feelings for the whole day.

#### 2.3.4. Cortisol Concentration and Perceived Stress

Women completed the Perceived Stress Scale [60] at baseline (*T* = 0 week), *T* = 4 weeks, and *T* = 16 weeks. In addition, morning salivary samples were collected at home on two different days prior to the beginning of the intervention (*T* = 0 week) and during the last two days of the 4-week intervention (*T* = 4 weeks) in order to measure cortisol concentrations. Cortisol is a reliable indicator of hypothalamic-pituitary-adrenal axis activity and is considered as a good indicator of altered physiological states in response to stressful situations [61]. Salivary cortisol reliably reflects the free fraction of cortisol in plasma [62] and is a noninvasive technique that can be used at home without interfering with the normal daily routine. In our study, participants had to take a sample immediately at the time of awakening as well as 15 minutes and 30 minutes thereafter. They were also told to avoid intense physical activity for two days prior to sampling and not to drink alcohol 24 h prior to sampling. The samples were kept refrigerated by the participant and were then frozen at −80°C until analyses were performed. Cortisol concentrations were determined by enzyme immunoassay (Salimetrics, Carlsbad, CA, USA) at CHU de Québec Research Center. An estimate of total cortisol secretion over the 30 min postawakening period was then computed as AUC for each day. A mean AUC was calculated in order to obtain an integrated score for the two days prior to the beginning (*T* = 0 week) of the intervention and for the last two days of the 4-week intervention (*T* = 4 weeks).

#### 2.3.5. Food and Energy Intake

A validated web-based self-administered food frequency questionnaire (FFQ) was completed at *T* = 0 and *T* = 16 weeks. The FFQ was validated within a Quebec City-based healthy population [63] and contains 136 questions separated into eight sections: dairy products, fruits, vegetables, meat and alternatives, cereals and grain products, beverages, “other foods,” and supplements. Participants were questioned about the intake frequency of different foods and drinks during the last month and could report the frequency of these intakes in terms of day, week, or month.

### 2.4. Statistical Analyses

Statistical analyses were performed using SAS software (version 9.3; SAS Institute, Cary, NC, USA). The distribution of all data was analyzed to ensure normality. Appropriate transformation was performed as needed to normalize the distribution of variables. Student's *t*-tests were performed to determine any between-group differences at baseline (*T* = 0 week). Mixed ANOVA procedures for repeated measurements were used to assess main effects of group, time, and group-by-time interaction. Mixed procedure allows using all available data at every time even if participants had missing data. Adjustment for differences at *T* = 0 week was systematically performed when needed (i.e., for variables that were significantly different between groups at *T* = 0 week as determined by Student's *t*-tests). When a significant main effect was detected, pairwise differences between and within group means were tested with the Tukey–Kramer adjustment. For all statistical analyses performed, an *α* level of 0.05 was used.

## 3. Results

**Figure 1:**
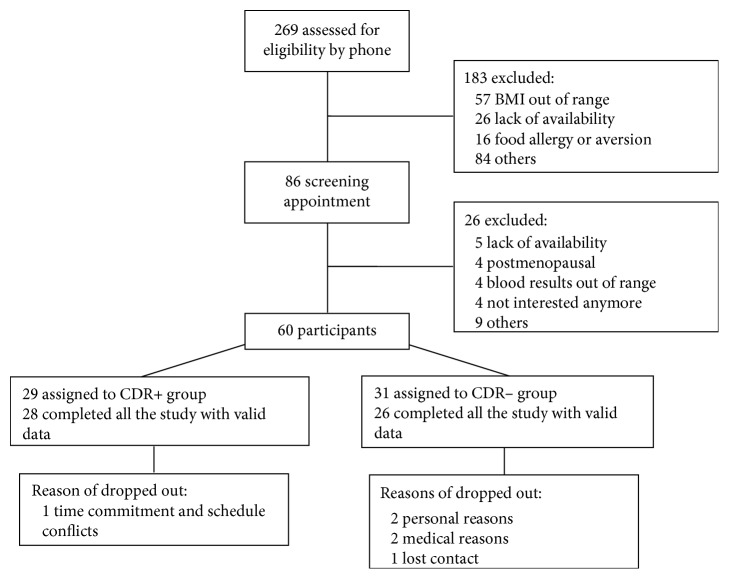
Flow diagram of subject's enrollment, assignment, and completion of the study protocol.

Eighty-six women met the initial inclusion criteria and were invited to a screening appointment (Figure 1). Of this number, 60 met all the inclusion criteria and were included in the study. Among this initial group, 4 participants dropped out during the 4-week intervention and 2 during the follow-up (1 in the CDR+ group and 5 in the CDR− group). Therefore, 54 participants completed the study (28 in the CDR+ group and 26 in the CDR− group). Compliance to the diet during the 4-week controlled feeding intervention was very good among participants (97.1% in CDR+ group; 97.7% in CDR− group) and was not significantly different between the two groups (*p*=0.4385).

### 3.1. Baseline Characteristics

Table 2 shows baseline characteristics (*T* = 0 week) of women from the CDR+ and CDR− groups. The whole sample of women had a mean BMI of 32.0 kg/m^2^, ranging from 25.8 kg/m^2^ to 50.2 kg/m^2^. RMR and energy needs to remain weight stable (as evaluated by the measurement of RMR and the activity factor) were not different between the two groups. There was a trend for a lower age in the CDR− group (*p*=0.0558). However, adjustments for this variable in subsequent analyses had no effect on the results. Therefore all data presented are not adjusted for age. No other significant differences were observed between the groups (*p* ≥ 0.1360).

### 3.2. Body Weight Change

**Figure 2:**
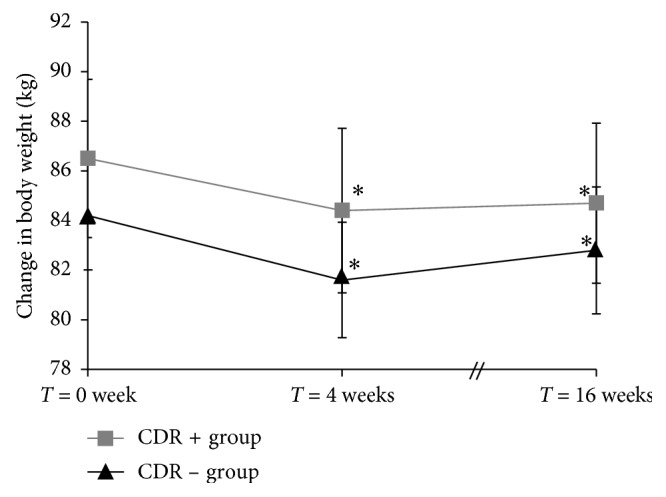
Mean (±std error) change in body weight for women from the CDR+ and CDR− groups over time. Analysis was performed on inverse transformed values. A significant time effect was observed (*p* < 0.0001). ^∗^Significant change from *T* = 0 week (*p* ≤ 0.0323). CDR+, with experimentally induced cognitive dietary restraint; CDR−, without experimentally induced cognitive dietary restraint.

There was an effect of time on body weight (*p* < 0.0001; Figure 2). As expected, body weight at *T* = 4 weeks was lower than at *T* = 0 week in both CDR+ and CDR− groups (mean weight loss: CDR+ group: −2.4 kg; CDR− group: −2.1 kg). The observed weight loss at *T* = 16 weeks was not different than at *T* = 4 weeks in both groups. There was no group-by-time interaction (*p*=0.6833). BMI and waist circumference also decreased with time (*p* < 0.0001), with no group-by-time interaction. More precisely, BMI and waist circumference decreased in both groups between *T* = 0 week and *T* = 4 weeks and then remained stable between *T* = 4 weeks and *T* = 16 weeks.

### 3.3. Eating Behaviors Questionnaires

At *T* = 0 week, CDR and flexible restraint were lower in the CDR+ group than in the CDR− group. Therefore, further analyses performed with these variables were systematically adjusted for the *T* = 0 week value. As shown in Table 3, a significant group-by-time interaction was observed for CDR (*p*=0.0037). As expected, while an increase in CDR was observed in women from the CDR+ group (*p* < 0.0001), no such change was observed in the CDR− group (*p*=0.9114). There was no other group-by-time interaction for any other eating behavior traits. However, some time effects were observed. Flexible restraint increased while disinhibition, hunger, and cravings decreased in response to the 4-week energy restriction (*p* ≤ 0.0016). A marginal increase in rigid control was also found (*p*=0.0594).

### 3.4. Appetite Score

**Figure 3:**
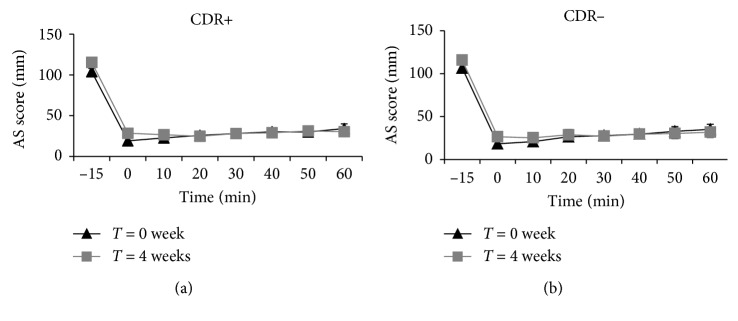
Appetite score values measured before and at different time points following the standardized breakfast for women from the CDR+ (a) and CDR− (b) groups. There is no group-by-time interaction at any time points. A significant time effect was found for AS before (−15 min) and immediately after (0 min) breakfast (*p*=0.0081 and *p*=0.0189, resp.). CDR+, with experimentally induced cognitive dietary restraint; CDR−, without experimentally induced cognitive dietary restraint.

Figure 3 shows AS values measured before, immediately after, and at 10, 20, 30, 40, 50, and 60 min after the consumption of the standardized breakfast. No group-by-time interaction was observed, meaning that both groups responded similarly to the intervention. However, a significant time effect was found in the fasting state (*p*=0.0081) and immediately after consumption of the breakfast (*p*=0.0189) with AS values being higher at *T* = 4 weeks than at *T* = 0 week. No group-by-time or time effect was observed for the 1 h AUC for AS or for the 1 h AUC for each appetite sensation evaluated separately.

An increase with time was found for desire to eat nonstudy foods (*p*=0.0157). In addition, women reported being more satisfied with the amount of food consumed with time (*p*=0.0015). No time effect was found for hunger sensation and no group-by-time interaction was observed for any of these variables.

### 3.5. Stress

All results related to stress are presented in Table 4. No group-by-time interaction was observed for any of the variables studied. However, a time effect was found for perceived stress which decreased with time (*p*=0.0002). No significant changes with time were found for cortisol AUC.

### 3.6. Dietary Intakes

Table 5 shows dietary intakes at baseline (*T* = 0 week) and at follow-up (*T* = 16 weeks). The only group-by-time interaction was for the intake of saturated fatty acids. In the CDR+ group, a decrease in the contribution (in percentage) of saturated fatty acids to total energy intake was observed whereas no such change was observed in women from the CDR− group. Time effects were found for energy intake as well as for intakes of fruits and vegetables and grain products (*p* ≤ 0.0049). Indeed, women decreased their energy intake as well as their consumption of grain products during the follow-up period compared to their preintervention values. An increase in the consumption of fruits and vegetables over the same period of time was also observed. Intakes of dietary cholesterol and sodium also decreased at follow-up compared to *T* = 0 week. Since in Quebec there are seasonal differences in food availability, we have verified whether changes in intakes from the different food groups were influenced by the season during which women reported their follow-up dietary intakes. These additional analyses showed that the season had no impact on reported intakes from the different food groups.

## 4. Discussion

Most studies of weight loss performed to date have been conducted in a context favoring both energy restriction and increased CDR. It is therefore difficult to untangle in these previous studies the respective contributions of energy restriction (i.e., homeostatic factors) and induced CDR (i.e., cognitive factors) on variables that can lead to weight regain. Accordingly, a strength of the present study was the robust well-controlled study design that allows to appropriately isolate the impact of experimentally induced CDR from the impact of energy restriction. In this study conducted in a sample of overweight and obese women, we found, contrary to our hypotheses, that inducing CDR in a context of energy restriction had no further effects on eating behavior traits, appetite sensations, and markers of stress in the short term as well as in the longer term than energy restriction alone. From our results, it could therefore be concluded that increasing CDR has no negative impact on factors regulating energy balance in the context of energy restriction. However, before generalizing such conclusion, some points first need to be addressed.

The low energy density of the experimental diet was necessary in order to ensure that women in the CDR− group did not detect the energy restriction, that could have led to an increase in CDR. However, we are aware that being exposed to this highly satiating diet could have attenuated in the CDR+ group the perceived deprivation effect that usually accompanies an increase in CDR. This hypothesis can be further supported by the observed decrease in women from both groups in the hunger score (as assessed by the TFEQ) in response to the intervention. Some studies have documented that a deprivation state is a contributor to food cravings and risk of disinhibition among highly restrained individuals [65–68]. The attenuation of perceived deprivation because of the satiating diet in the present study could have thus lessened the impact of the increase in CDR on eating behavior traits and appetite sensations. Furthermore, some studies have suggested that food cravings and risk of disinhibition were more closely related to rigid control than flexible control [52, 69]. In our study, while CDR increased in women from the CDR+ group, rigid control did not increase significantly. Results of the present study cannot rule out the possibility that increased rigid control may have deleterious effects on cravings and disinhibition in a weight loss process.

The well-controlled context used in our study was needed to ensure a similar caloric restriction in both groups. However, it is possible that this controlled context may have lessened the preoccupation over food choices in women from the CRD+ group. According to previous studies that have shown an association between concerns about food and diet and CDR [70], the relatively low preoccupation about food in the present study could have prevented a larger increase in CDR than the one actually observed in traditional weight loss studies. Accordingly, other studies that have addressed the impact of the change in CDR on weight loss or eating behaviors have observed greater increases in CDR score than the one achieved in the present study [18, 71]. Furthermore, because the initial CDR score of the CDR+ group was significantly lower than the CDR− group, added to the fact that the increase in CDR was relatively small, this led at the end of the 4-week intervention to a CDR value in the CDR+ group that was not significantly higher than the initial CDR score of the CDR− group (resp. 8.5 versus 8.2). We can speculate that reaching higher CDR values, which are typical of weight loss interventions, could have led to different outcomes.

Some studies have linked CDR and trying to lose weight with increased stress and cortisol concentration [27, 72]. According to those past observations, we could have expected an increase in stress at *T* = 4 weeks in the CDR+ group. However, we found no significant change in cortisol concentration whereas perceived stress decreased in both groups in response to the intervention. This could be in part related to the absence of concern about monitoring food intake in our study design. Indeed, Tomiyama et al. have shown that perceived stress increases only among individuals who monitor their caloric intake via a food diary while individuals who are only subjected to caloric restriction show no significant change in their score. In fact, contrary to a real-life setting where individuals need to put lots of effort in controlling food intake (e.g., counting calories, limiting some foods, and reducing portion size), women in our trial did not have to make any food choice for a period of 4 weeks. Thus, according to the results obtained by Tamiyama et al., this absence of concern for recording daily intake could explain why perceived stress did not increase in our study.

Since the controlled context of the intervention might have lessened the impact of increasing CDR on variables that can lead to weight regain, we could have expected that during the 3-month follow-up, some between-group differences would emerge because women had to make their own food choices. However, no significant between-group differences were obtained at the 3-month follow-up for eating behavior traits, body weight, and perceived stress. One potential explanation for this absence of difference is that being exposed for 4 weeks to low energy density foods might have encouraged women to modify their eating habits and continue to choose low energy density food during the follow-up. Accordingly, it was found that women from both groups improved their eating habits during the follow-up period compared to their baseline intakes. In fact, in both groups, a significant increase in fruit and vegetable consumption was observed and significant decreases in sodium and cholesterol intakes were also found. Furthermore, although only women from the CDR+ group received advice to decrease energy intake, both groups reported a similar reduction in energy intake. The instructions of increasing fruit and vegetable consumption and of focusing on low energy density food provided in the CDR– group may explain the decrease in energy intake in this group. Overall, changes observed in food choices during the follow-up suggest that being exposed to a highly satiating diet along with some dietary recommendations favor the adherence to a healthier food pattern. This observation is concordant with results from a study conducted by our group in which it was shown that being exposed to a satiating Mediterranean diet in a controlled context was associated with improvement in dietary habits in the longer term [73].

Strengths of the current study include the study design and the rigorous measures used throughout the project. The highly controlled nature of the intervention allows a similar weight loss to be reached in both groups, which suggests that the energy restriction was similarly implemented in both groups as planned. In addition, the experimental manipulation of CDR was also performed as planned since only women in the CDR+ group experienced an increase in CDR. However, this study also has some limitations. The facts that compliance was assessed by self-report and that food intake at baseline and at the follow-up was evaluated by a FFQ, which is known to be prone to some memory bias and underreporting, can be considered limitations. In addition, even if the validated FFQ used in the present study contains main foods consumed in Quebec, this tool considers only a finite number of foods, which may also lead to underreporting. The fact that we observed a weight maintenance during the follow-up despite reported energy intakes at 16 weeks much lower than calculated energy needs may indeed be suggestive of an underreporting of energy intake. We would also like to underline that although the small increase in CDR score induced in the present study was not associated with any detrimental changes in variables influencing energy balance, these results cannot be generalized to conditions where the increase in CDR would be much larger as it is the case in traditional weight loss approaches. Moreover, the experimental design of this study may have attenuated the perceived deprivation effect that usually accompanies CDR in the CDR+ group, limiting the impact of CDR on eating behavior traits, appetite sensations, and stress. Therefore, it is clear that further studies will be needed to put some lights about the impact of increasing CDR on these variables that can lead to weight regain.

## 5. Conclusion

Results of the present study suggest that a small increase in CDR in the context of a fully controlled-energy-restricted diet with high satiating properties has no detrimental effects on eating behavior traits, appetite sensations, and perceived stress. Since our study is the first to investigate the difference between a state of energy restriction alone and a state of energy restriction combined to experimentally induced CDR on factors regulating energy balance, these results represent an interesting addition to the current literature in order to shed light about the place that increasing CDR could take in weight management. In addition, these results support the relevance of using highly satiating diet for body weight management. Further studies should continue to explore the associations between CDR and factors that can favor body weight regain in weight loss studies performed in different settings. It would be especially relevant to tease out the impact of the increase in CDR in response to a weight loss intervention versus the absolute level of CDR reached following such an increase on factors that influence body weight control.

## Figures and Tables

**Table 1:** Composition of the 4-week controlled diet.

Variables	Value
Carbohydrate (% energy)	50.4
Protein (% energy)	17.4
Fat (% energy)	32.2
Fibers (g/10,000 kJ)	43
Energy density (kJ/g)	3.8

**Table 2:** Baseline characteristics (*T* = 0 week) in CDR+ and CDR− groups^a,b^.

	CDR+ *n*=29	CDR− *n*=31
Age (years)	41.1 ± 6.3	37.7 ± 6.9
Body weight (kg)^†^	86.5 ± 17.2	84.2 ± 12.2
Height (m)	1.64 ± 0.06	1.63 ± 0.06
BMI (kg/m^2^)^†^	32.4 ± 6.5	31.7 ± 4.1
Waist circumference (cm)	99.6 ± 12.6	98.5 ± 9.1
RMR (kJ/day)	7,004 ± 1,036	6,626 ± 756
Energy needs (kJ/day)	10,978 ± 1,702	10,417 ± 1,369

CDR+: with experimentally induced cognitive dietary restraint; CDR−: without experimentally induced cognitive dietary restraint; RMR: resting metabolic rate. ^a^Baseline values did not differ significantly between groups (unpaired *t*-test). ^b^All values are mean ± SD. ^†^Analysis was performed on inverse transformed values.

**Table 3:** Eating behavior traits at each time point for women from the CDR+ and CDR− groups^a^.

	CDR+ (*n*=29)			CDR− (*n*=31)			*p*		
	*T* = 0 week	*T* = 4 weeks	*T* = 16 weeks	*T* = 0 week	*T* = 4 weeks	*T* = 16 weeks	Group	Time	Group ∗ time
CDR^b,c,†^	5.6 ± 3.5	8.5 ± 4.4	7.5 ± 4.3	8.2 ± 4.3	8.7 ± 4.4	8.7 ± 4.1	0.0167	<0.0001	0.0037
Flexible control^b,c^	1.7 ± 1.4	2.7 ± 1.9	2.7 ± 1.9	2.7 ± 2.0	3.1 ± 1.8	2.9 ± 1.8	0.2611	0.0006	0.2077
Rigid control^c^	1.9 ± 1.4	2.6 ± 1.7	2.4 ± 1.6	2.2 ± 1.6	2.3 ± 1.5	2.4 ± 1.6	0.8311	0.0594	0.2557
Disinhibition^c^	7.6 ± 2.6	5.8 ± 2.8	6.7 ± 2.6	8.3 ± 3.3	6.1 ± 3.1	7.3 ± 3.4	0.3899	<0.0001	0.8925
Hunger^c^	6.7 ± 2.9	5.5 ± 3.2	4.6 ± 2.6	5.5 ± 3.2	4.2 ± 2.6	4.7 ± 3.5	0.3094	0.0016	0.2539
Cravings^d^	104.7 ± 31.5	94.1 ± 26.6	94.5 ± 27.9	109.4 ± 33.9	92.6 ± 28.5	93.3 ± 33.2	0.8467	<0.0001	0.6111

CDR+: with experimentally induced cognitive dietary restraint; CDR−: without experimentally induced cognitive dietary restraint. ^a^All values are means ± SD. ^b^Baseline values (*T* = 0 week) differed significantly between groups. Adjustment for baseline value was performed in analyses. ^c^Score from the Three-Factor Eating Questionnaire (TFEQ). Possible range of values is: 0 to 21 for CDR; 0 to 7 for flexible control and rigid control; 0 to 16 for disinhibition; 0 to 14 for hunger. ^d^Score from the Food Craving Questionnaire. Possible range of values is 39 to 195. ^†^Analyses were performed on log transformed values.

**Table 4:** Perceived stress and cortisol concentrations in women from the CDR+ and CDR− intervention groups^a^.

	CDR+ (*n*=29)			CDR− (*n*=31)			*p*		
	*T* = 0 week	*T* = 4 weeks	*T* = 16 weeks	*T* = 0 week	*T* = 4 weeks	*T* = 16 weeks	Group	Time	Group ∗ time
Perceived stress	14.2 ± 4.9	11.7 ± 6.3	11.6 ± 5.6	15.6 ± 5.5	13.9 ± 6.4	14.1 ± 5.1	0.1571	0.0002	0.6845
AUC of salivary cortisol (nmol/L/min)^†^	320.0 ± 140.7	322.8 ± 184.8	—	342.1 ± 168.3	328.3 ± 154.5	—	0.6815	0.6083	0.6309

CDR+, with experimentally induced cognitive dietary restraint; CDR−, without experimentally induced cognitive dietary restraint; AUC, area under the curve. ^a^All values are means ± SD. ^†^Analyses were performed on log transformed values.

**Table 5:** Dietary intakes in women from the CDR+ and CDR− groups^a,b^.

	CDR+		CDR−		*p*		
	*T* = 0 week (*n*=29)	*T* = 16 weeks (*n*=28)	*T* = 0 week (*n*=31)	*T* = 16 weeks (*n*=26)	Group	Time	Group ∗ time
Energy (kJ)	10,326 ± 2,992	9,226 ± 3,185	9,825 ± 2,662	8,856 ± 2,721	0.5516	0.0049	0.8273
Fruits and vegetables^†^	7.1 ± 2.9	8.9 ± 4.4	5.8 ± 2.3	8.0 ± 4.6	0.1391	0.0004	0.4699
Dairy products	2.6 ± 1.4	2.2 ± 1.2	2.4 ± 1.2	2.5 ± 1.4	0.7938	0.2938	0.0689
Meat and alternatives	3.2 ± 1.1	2.9 ± 1.1	2.9 ± 0.9	2.7 ± 1.0	0.2895	0.0698	0.6017
Grain products	5.5 ± 1.9	4.5 ± 1.5	5.6 ± 2.0	4.9 ± 1.9	0.4809	0.0002	0.8111
Cholesterol (mg)	345.6 ± 153.4	280.9 ± 101.4	298.4 ± 101.3	264.3 ± 101.7	0.2563	0.0016	0.2669
% total fat	36.8 ± 5.6	34.4 ± 5.2	35.9 ± 4.6	35.9 ± 5.1	0.8371	0.0921	0.1322
% saturated fatty acids	13.0 ± 2.2	11.5 ± 2.1	12.1 ± 2.1	12.1 ± 2.4	0.7357	0.0171	0.0303
Sodium (mg)	3,496 ± 928	3,082 ± 996	3,429 ± 1,026	3,075 ± 976	0.9979	0.0032	0.6375

CDR+: with experimentally induced cognitive dietary restraint; CDR−: without experimentally induced cognitive dietary restraint. ^a^Results were obtained with a validated FFQ. For food groups, results are presented as the mean number of portions per day according to Canadian food guide portion sizes for fruits and vegetables, dairy products, meat and alternatives, and grain product intakes. No data were presented at *T* = 4 weeks since dietary intakes were fully controlled during the 4-week intervention. Outliers were excluded using the Outlier Labeling Rule with a 2.2 interquartile range (IQR) multiplier [64]. ^b^All values are means ±SD. ^†^Analyses were performed on log transformed values.

## Supplementary Materials

Supplementary Materials Table S1: the 7-day cyclic menu used during the controlled diet intervention.
