# Supplementary material for: Impact of Experimentally Induced Cognitive Dietary Restraint on Eating Behavior Traits, Appetite Sensations, and Markers of Stress during Energy Restriction in Overweight/Obese Women
Source: J Obes. 2018 Jun 25;2018:4259389. doi: 10.1155/2018/4259389 (PMC6079586; doi:10.1155/2018/4259389)
Supplement: Supplementary Materials — Table S1: the 7-day cyclic menu used during the controlled diet intervention. [file 4259389.f1.pdf]

Table S.1: The 7-day cyclic menu used during the controlled diet intervention

Meals	Monday	Thuesday	Wednesday	Thursday	Friday	Saturday	Sunday
**Breakfast**	Müslix cereal	Whole grain bread	Raisin bran muffin	Whole wheat bread and margarine	Müslix cereal	Whole wheat bread and margarine	Ham and cheese whole weat english muffin
	Milk	Cottage cheese	Strawberry and orange smoothie	Hard boiled egg	Milk	Omelet	Cantaloupe
	Honey dew melon	Cantaloupe	Mozzarella cheese	Honeydrew melon	Pineapple	Green grapes	Grapefruit juice
	Almonds	Peanut butter and jam		Strawberry yogurt	Almonds	Jalsberg cheese	Strawberry yogurt
**Lunch**	Shrimp rice	Shepherd's pie	BBQ chicken	Ham sandwich	Frittata	Mushroom chicken	Chilli con carne
	Green salad and vinaigrette	Green beans	Peppers and zucchini	Coleslaw	Green salad and vinaigrette	Green beans	Raw vegetables
	Red grapes	Lentil soup	Rosted root vegetables	Vegetable juice	Whole wheat bread	Roasted potatoes	Ranch dip
		Apple and raisin cake	Leek cream	Apple sauce	Vegetable soup	Orange	Couscous
			Fruit salad		Fruit salad		Apple and berry sauce
**Dinner**	Chicken cacciatore	Tuna pasta	Greek tilapia	Pasta primavera	Salmon loaf	Veal meatball and vegetables	Pork
	Brocoli	Vegetable salad	Carrot	White kidney bean salad	Bechamel sauce	Tomato soup	Green peas
	Carrot soup	Pear	Spinach salad	Kiwi	Broccoli and cauliflower salad	Brown rice	Basmati rice
	Roasted potatoes		Bulgur		Vegetables and lemon orzo	Raspberry cake	Cauliflower soup
	Vanilla pudding		Green grapes		Date squares		Blueberry pudding
